# Improving Women's Health through Universal Health Coverage

**DOI:** 10.1371/journal.pmed.1001580

**Published:** 2014-01-06

**Authors:** Jonathan Quick, Jonathan Jay, Ana Langer

**Affiliations:** 1 Management Sciences for Health, Cambridge, Massachusetts, United States of America; 2 Department of Global Health and Social Medicine, Harvard Medical School, Boston, Massachusetts, United States of America; 3 Women and Health Initiative, Maternal Health Task Force, Harvard School of Public Health, Boston, Massachusetts, United States of America

## Abstract

Jonathan Quick and colleagues discuss how women's health world-wide can be improved through universal health coverage.

*Please see later in the article for the Editors' Summary*

**Summary Points**

- Unequal access to health care contributes to shortcomings in women's health at all ages. The post-2015 United Nations development framework must address these inequalities globally.
- Universal health coverage (UHC) is considered a leading candidate among health-related targets. It is the one approach that reduces inequitable access and addresses the full range of women's health issues with the full spectrum of health services.
- UHC has proven a powerful driver for women's health in low- and middle-income countries including Afghanistan, Mexico, Rwanda, and Thailand. Success requires a gender-sensitive approach to design and implementation around (1) the essential services package, (2) improving access to services, (3) eliminating financial barriers, (4) reducing social barriers, and (5) performance monitoring.
- To expand coverage and effectively deliver quality services to all women, health systems must become stronger around leadership, management, financing, human resources, community involvement, and other critical elements.
- Essential measures of UHC include women's access to health care, coverage equity for essential services for women, financial protection for women and impact on women's health outcomes. Post-2015 UHC indicators should retain the Millennium Development Goals' focus on priority health outcomes.
- Women's health must be a shared agenda for which success requires active engagement by country political and health leadership; civil society, including advocates for women's health, sexual, and reproductive health rights; multilateral agencies; global health funders; and all others concerned with women's health and equity.

With the Millennium Development Goals (MDGs) expiring in 2015, the world awaits a new, ambitious framework for improving lives. This framework must pass the vital test of reducing inequalities, especially gender-related ones, as a critical step towards the improvement of women's health. Measurably improving women's health throughout the life course will contribute to other post-2015 goals. Conversely, women's social empowerment through education, participation in the labor market, and political representation will improve health.

For this virtuous cycle to occur, the next iteration of the development goals has to embrace women's health and wellbeing as a key priority, particularly since indicators for women's health in the current MDGs, including maternal mortality, are among those lagging farthest behind [1]. Health systems have failed to provide contraceptive access to many women who wish to delay or avoid pregnancy, especially for poor women [2]. Slow progress also reflects a failure to address complications of pregnancy and childbirth, such as life-threatening hemorrhage and hypertension. The ongoing policy debates around the post-2015 development agenda provide a unique window of opportunity to redress these limitations and advance a comprehensive women's health agenda.

There is an emerging consensus that universal health coverage (UHC) is an essential means of achieving post-2015 health goals and may represent a valuable goal or target in itself [3]–[5]. UHC, according to the World Health Organization (WHO), is the goal that all people can obtain the prevention and treatment health services they need without suffering financial hardship when paying for them [6]. Supported by growing evidence [7], proponents argue that UHC can improve population health and promote economic development by lifting the barriers created by unequal access to quality health care services.

To deliver on this promise, UHC efforts must be designed, implemented, and measured appropriately. In this article we analyze the connection between UHC and the women's health agenda, recommending policy measures that can help to ensure that adoption of UHC, as part of the post-2015 framework and implementation of UHC in national health systems, will directly contribute to improving women's health.

## UHC Efforts in Low- and Middle-Income Countries

Countries employ different mechanisms in their efforts to achieve UHC. Beyond the vision of improving health care access and reducing financial hardship, their approaches share common traits. There is a characteristic transition from individual out-of-pocket expenditure towards prepayment and risk pooling, often through a new mechanism such as a national health insurance scheme [8]. Government subsidies support those who cannot afford premiums. Increased subsidies are made possible through greater government expenditure on health, often using revenue from new taxes, international assistance, and other mechanisms.

For governments, stewardship over pooled funds provides a “control knob” [9] to rationalize service delivery, using predefined benefits packages and more efficient delivery systems. This capability will be a major step forward in countries where spending is mostly out-of-pocket and health systems are weak. Typically, UHC programs define a basic package of health services, which every enrollee can expect to receive. Determining this package is a key component of UHC design. The UHC movement has promoted, as its first priority, making vital primary care services available to everybody, especially the most vulnerable members of society, which usually include women and children. This approach is most equitable and appears most effective for reducing preventable mortality [10].

Financing and service delivery reforms will differ according to a country's level of existing health system, economic development, and other factors. But progress toward UHC is possible everywhere. Evidence supports the effectiveness of UHC reforms for improving financial protection and increasing the utilization of key health services (such as antenatal care), especially among poor people [7].

## Inequities in Women's Health

Inequities affecting women's health have been widely documented throughout the entire life course from the girl child to the older woman [11],[12]. An extensive WHO analysis of women and health confirms, for example, the extent of deaths from unsafe abortion, the limited progress in reducing unmet need for family planning in sub-Saharan Africa, and the reality that roughly 80% of cervical cancers occur in countries where prevention, screening, and treatment are limited or non-existent [11].

In a Countdown to 2015 study of 54 countries, Barros et al. [13] found that among 12 maternal and child health indicators, the three with the greatest income-related inequity were all for women's health services. Coverage of skilled birth attendants was the least equitable, with a mean coverage of 32.3% versus 84.4% for the poorest and richest quintiles, respectively. This indicator was followed by four or more antenatal visits (35.9% versus 70.5%), then family planning needs satisfied (41.4% versus 67.0%). In a related study, Victora and colleagues [14] found that rapid increases in antenatal coverage (as might be seen with UHC) was associated with improved equity.

## Addressing Health Care Inequalities through UHC

Women, children, and others most visibly affected by health care inequalities stand to gain the most from well-designed UHC programs. First, UHC removes financial barriers such as user fees at the point of service, reducing burdens on poor people, and especially women, who often have primary responsibility for their families' health care but lesser access to cash.

Second, while UHC efforts do not guarantee universal access to every possible health care service, they typically provide at least a basic set of high-impact primary care interventions to all users. Basic services packages can and usually do provide core reproductive and maternal health services, including necessary interventions for safe, effective contraception and the basic services proven to prevent the vast majority of maternal deaths [15]. In Afghanistan, a low-cost basic services package implemented in 2003 (see Box 1) consisted almost entirely of interventions for family planning and addressing the most common causes of maternal and child mortality [16].

**Box 1. Expanding Coverage in Afghanistan**

The Afghan health system demonstrates how a UHC approach can prioritize key women's health services, even in challenging settings. In 2003, the Afghan Minister of Public Health, Dr. Suhayla Seddiqi, announced a move towards UHC that rolled out a nationwide essential benefits package. It emphasized basic primary care interventions like those for family planning and maternal health [16], largely delivered by a new cadre of community health workers and community midwives [33]. Coverage improved: between 2003 and 2010, women's contraceptive use doubled, from 10% to 20%, and access to skilled antenatal care increased from 16% to 60% [34]. Though maternal mortality is still high (approximately 300–400 deaths per 100,000 births), it has dropped two-thirds since 2002, and is lower than would be predicted for a country where three-quarters of women receive no education [35]. If UHC had been complemented with effective efforts to reduce poverty and conflict, and improve women's rights, the progress would have been undoubtedly even greater.

*Afghanistan Basic Package of Health Services, focus areas:*

- child immunization
- micronutrient supplementation and nutrition screening
- tuberculosis and malaria control
- prenatal, obstetrical, and postpartum care
- family planning
- basic curative services, including integrated management of childhood illnesses

Third, women's interest in health care extends far beyond their own maternal and reproductive health needs. Comprehensive care promotes good health for women and their families throughout their lives, from prevention of low birth weight and prematurity, to childhood nutrition and immunization, to prevention and treatment for chronic conditions such as heart and lung disease, mental health disorders, diabetes, and cancers. Services provided in the context of UHC become more user-friendly as they are integrated—available from the same provider or under the same roof. Equitable access to integrated, comprehensive care is more likely in UHC-style systems, which enable policymakers and health planners to rationalize service delivery.

## Designing and Implementing UHC That Works for Women

Many low- and middle-income countries are already pursuing UHC [17]. Making UHC a central focus of the post-2015 development framework (discussed below) will encourage many more nations to join these efforts.

Well-designed UHC programs have shown positive impacts on women's access to needed health services. In 2003, *Seguro Popular* (Popular Health Insurance)—a core element of Mexico's commitment to UHC—was established to dramatically expand population coverage for 90% of the common health conditions. Within its first two years, the new program was associated with increases in several priority interventions, including four of specific relevance to women: skilled birth attendance, antenatal care, cervical cancer screening, and mammography [18]. In-depth analyses published nine years later, in 2012, concluded that the program was “improving access to health services and reducing the prevalence of catastrophic and impoverishing health expenditures, especially for the poor” [19] and generating a wide range of positive impacts on women's health [20].

Thailand's 2001 UHC program expanded access to nearly 100% of the population for comprehensive benefit package, which included a wide range of maternal, sexual, and reproductive health services. Equitable public financing reduced out-of-pocket spending and virtually eliminated medical impoverishment. A national study conducted in 2005–2006 found a negligible gap between rich and poor in prenatal care, delivery care, and family planning [21]. In 1999, Rwanda established community-based health insurance (Mutuelles de santé) as part of its strategy for UHC [22].

These and other experiences indicate that for UHC to measurably improve health and equity for women, programs must address five critical factors in their design and implementation: (1) essential services package, (2) access to services, (3) financial barriers, (4) social barriers, and (5) performance indicators (Table 1).

**Table 1:** Critical factors for designing and implementing UHC to improve women's health.

Factor	Challenges for Women's Health and Equity	Critical Actions for UHC Design and Implementation
1. Essential services package	Service packages often lack elements essential for women's health [23]. For example:• An early study of public essential service packages in 152 countries found only 20 included all expected maternal and reproductive services.• Commonly absent services were delivery care, emergency obstetric care, and safe abortions even where legal.• Adolescent girls and older women are often excluded due to focus on maternal health.	Incorporate all essential services for women throughout the life course, including:• Contraception and reproductive health services.• Antenatal care, skilled birth attendants, emergency obstetrical care, post-partum care, other elements of safe motherhood.• Preventing, screening, treatment for breast, cervical, other women's cancers.• Abortion services, where legal.
2. Access to services	Especially for women with multiple work, household, family responsibilities access may be reduced by:• Separation of services in different facilities or on different schedules (e.g., prenatal care, childhood immunizations, AIDS treatment).• Physical distance and hours of service create barriers for women with household and family responsibilities.	Promote convenient, close-to-home services by, for example:• Integrating services to the maximum extent to allow “one stop” services for women, children.• Combining AIDS screening with confirmation and initiation of treatment.• Legalizing and reimbursing contraception provided by pharmacies, licensed drug sellers.• Addressing health workforce shortages, including scaling up community-level providers who can deliver essential services for women.
3. Financial barriers	• OOP payments are generally greater for women than for men [35].• Higher OOP payments by women contribute to greater unmet health needs among women [36].	• For insurance-based UHC, subsidize premiums to ensure coverage for women.• Eliminate or minimize OOP payments for priority women's health services.
4. Social and other non-financial barriers	Social and other non-financial barriers to care may include:• Lack of female health providers, provider attitude.• Cultural or legal expectations for husband or parental permission for care.	Systematically monitor women's access to services across locally relevant dimensions, which may include cultural, health provider, and other factors [37].
5. Performance monitoring indicators	• Excessive focus on indicators of financing, OOP payments, and impoverishment may overshadow indicators of service delivery and health outcomes.	As illustrated in Table 2, UHC monitoring indicators should include:• All priority women's health services and health outcome measures.• Measures of equity in delivery of women's health services.• Gender disaggregation of service and health outcome information.

OOP, out-of-pocket.

Maximizing the benefits of UHC for women also requires strengthening health systems at multiple levels, including financing, human resources, and community involvement. Poor design can reinforce gender inequities, with women falling through the cracks of patchwork insurance schemes, and too narrow a range of reproductive health services available [23]. Low quality of care, often overlooked as services are scaled up, can undermine health impact even when coverage appears to have increased. When improving health systems, women, who play multiple and essential roles as leaders and health care givers in the informal and formal health systems, must be recognized as a key part of the solution and drivers of positive change [24].

Delivering more services requires more service providers. The move towards UHC requires improvements in health workforce recruitment, training, deployment, management, and retention. Community health workers and community midwives can cost-effectively scale up essential women's health services, such as family planning, antenatal care, and delivery attendance, especially in poor and rural settings. However, their effectiveness and sustainability depends on thoughtful management, with particular attention to the challenges women face as providers and users in these settings.

Similarly, UHC success depends on the availability and management of essential medicines. Medicines, along with micronutrient supplementation and other life-saving commodities, are central to maternal health, contraception, and other key women's health services. Making these products widely available requires a strong supply chain and trained providers. Effective pharmaceutical management, from procurement to the provider level, is necessary to ensure safety and appropriate use, and to prevent medicines costs from overwhelming UHC budgets [25].

## Measuring Impact

The MDGs adopted by the UN in 2000 expire in 2015. The MDGs contain three health goals (child mortality, maternal mortality/family planning, and communicable disease) and a gender equality goal. The health MDGs have been credited with focusing international efforts around priority areas, but criticized for reinforcing a vertical (disease-specific) approach and for failing to incorporate equity, which may have incentivized policymakers to focus on the most easily accessible populations.

In the framework that will replace the MDGs in 2015, it is expected that there will be only one health goal. Priorities include continued progress on the MDG health agenda and new attention to the growing burden of disease from chronic conditions like cancers, heart and lung disease, and diabetes. WHO and others have considered UHC as an overarching health goal that could incorporate all priorities by improving affordable access to health care services [26]. Others have proposed UHC as a sub-goal alongside disease-specific outcomes target [5].

Both in the post-2015 framework and in country-level policies, some constituencies, including women's health advocates, consider UHC a means and not an end in itself. They worry that a UHC goal would be too detached from real-world priorities, measured only by indicators such as service coverage and financial protection, and would divert attention and resources from actual health outcomes, such as maternal and reproductive health. This pitfall can be avoided by tying the UHC process to specific health impact indicators and service delivery measures. Whether health outcomes stand alongside UHC (as recommended by the Sustainable Development Solutions Network) or fall beneath a UHC “umbrella” in the post-2015 framework, it should be understood that UHC programs are ultimately judged by their impact. At the country level, policymakers should tailor UHC benefits packages to address these specific health targets, then monitor and evaluate progress against them.

Among multilateral organizations such as the World Bank and WHO, a push is underway to develop indicators for UHC, especially for potential post-2015 use. National health leaders, academics, civil society (especially advocates for women's health and reproductive rights), and other stakeholders should actively engage in this process. For women's health, the framework should retain key indicators including maternal mortality ratios, adolescent birth rate, contraceptive prevalence rate, and unmet need for family planning (see Table 2). A UHC indicator associated with contraceptive coverage sends a powerful message about reproductive health's essential place in any UHC scheme. Ambitious targets should also reflect our ability to improve reproductive health by scaling up low-cost, high-impact interventions; these include antenatal care, use of skilled birth attendants, and provision of micronutrient supplementation and other life-saving commodities [27] and essential medicines [28] for reproductive health along the continuum of care [29]. For conditions that affect women but are not specific to women, indicators and data collection must disaggregate by sex to highlight gender inequalities in health. Coverage inequality must be carefully defined and systematically assessed [30].

**Table 2:** Examples of key service delivery and health outcome indicators for women's health.

**Service delivery indicators**
• Contraceptive prevalence rate
• Unmet family planning needs
• Antenatal care coverage
• Deliveries with skilled birth attendant
• Access to life-saving commodities and essential medicines along continuum of reproductive health care
**Health outcome indicators**
• Maternal mortality ratio
• Adolescent birth rate
**Equity indicators**
• Coverage inequality for antenatal, skilled birth attendant, and family planning coverage (ratio and absolute differences for poorest versus richest quintiles [13]
• Gender disparities in priority health outcomes (e.g. NCD, HIV/AIDS, TB, malaria)
• Gender disparities in impoverishment/financial protection for health

Policy responses to gender-based health inequalities must be comprehensive and multi-sectorial—in maternal health, for example, the unfinished agenda will only be advanced when gender gaps are narrowed, social determinants of poor maternal health are successfully addressed, and neglected aspects of maternal health, such as morbidities and their links with conditions in pre- and post-reproductive stages, are incorporated [31]. Social issues must be recognized within UHC frameworks, while other sectors, such as education and employment, must target health improvement [32]. Women's health should be understood as a priority cutting across the entire post-2015 framework.

## UHC and Women's Health: A Shared Agenda

As examples from Afghanistan, Mexico, Rwanda, Thailand, and other countries demonstrate, commitment to UHC can be a powerful driver to improve health outcomes and equity for women. Priority health objectives can—and must—guide the design and execution of UHC strategies. Both within countries and at the global level, the goal of achieving UHC can help expand coverage, align services, and accelerate equitable progress towards improving women's health.

Women's health must be a shared agenda, with active engagement from country political and health leaders; local and international civil society, including advocates for women's health, sexual, and reproductive health rights; multilateral agencies; global health funders; and all others concerned with women's health and equity. We urge groups that already support UHC principles of equity, access, and affordability to embrace the UHC concept. The global health community must ensure that women's health priorities are fully represented in UHC schemes, as well as the post-2015 health framework.

The strategies discussed in this article—including the importance of a comprehensive essential benefits package for women, attention to reducing or eliminating financial and social barriers to women's health care, and inclusion of service delivery and health outcome indicators—should provide the focus for designing and implementing UHC that works for women. These strategies can also guide advocacy for women's health and UHC, which must accelerate rapidly to influence the post-2015 agenda.
